# The Management of Dental Practice

**Published:** 1892-02

**Authors:** Louis Jack

**Affiliations:** Philadelphia


### International Dental Journal.

Vol. XIII. February, 1892. No. 2.

### Original Communications.[1]

### THE MANAGEMENT OF DENTAL PRACTICE.[2]

BY LOUIS JACK, D.D.S., PHILADELPHIA.

Mr. President and Members of the New York Odontological Society,—The chairman of your Executive Committee requested me to consider before you the topic which has been announced. This request must be the excuse for directing your attention to means and methods which have been peculiar to the conduct of my practice. It is on his assurance that the discussion of this subject may be of service that a natural reluctance to describe such matters has been overcome. While what is to be related to you will probably be of little benefit to those of established habits, it may prove of use to the younger men who are advancing to the stage of full practice. If this should be the result, the end will justify the means. The matter will be presented in the simplest and most direct manner.

It does not fall within the province of this address to consider the value upon practice of the fundamental requisites to secure professional success,—viz., professional training; general intelligence; moral rectitude; courtesy of manner, or personal neatness.

[1] The editor and publishers are not responsible for the views of authors of papers published in this department, nor for any claim to novelty, or otherwise, that may be made by them. No papers will be received for this department that have appeared in any other journal published in the country.

[2] Read at a meeting of the New York Odontological Society, November, 1891.

Without the combined possession of these characteristics there is little probability of any one securing a practice which would require methodical control. These elements are all so essential that at any stage of practice the loss of any of several may impair the career of any one.

One may, however, possess these qualities and yet not secure an adequate and deserved support. He may fail to make an impression upon his community for the lack of methodical arrangement of means conducive to the encouragement of patronage. Or the demands for service may, on the other hand, become so great that, for the lack of plans to utilize time and to control the demand, he may be strangled, as it were, by the abundance of patronage.

It is to the latter condition that I shall mostly direct your attention, because I believe the means which are best calculated to control an overpressure of practice are those which will prove most valuable to any one having the qualifications to entitle him to support, to secure the result each should aim for,—a life of effective and continued usefulness to his fellow-men.

The practice of dentistry is one requiring the closest attention to the duties involved in it by him who has assumed it. His office is to alleviate and prevent disease, and he cannot turn backward after he puts his hand to the plough. The obligation to stand ready to satisfy the demands made upon those engaged in dental practice are as imperative as are the obligations of the general physician.

Since the labor is exacting and makes the most severe strain upon the nervous forces of even the healthiest of men, it becomes every one to consider those matters which in any way may reduce this tension and maintain a state of constant efficiency.

Dental practice as usually pursued has not sufficiently regarded those economies of effort and arrangements of time which I am about to commend.

In common with others, I commenced practice by attending to the cases as they were presented; at length, as practice increased, I became hopelessly bound to conditions which overpowered me. To face the facts of strained health, deterioration of the quality of the service, and threatened reduction of income required the adoption of some system for the alleviation of these conditions.

This became established as follows:

*a.* In the arrangement of fixed hours for labor.

*b.* The avoidance of interruption, by refusing to receive calls or have consultations except at a certain hour of each day.

*c.* The distribution of the appointments evenly throughout the working months of the year.

*d.* The orderly arrangement of engagements for the care of the teeth, by which the distribution of time was effected, and also thereby to limit as far as possible the occurrence of the severe and protracted operations.

*e.* And at length the employment of a secretary to carry out these arrangements.

The first proposition is so manifestly correct that it needs little elucidation to enforce it. It is clear that the exhaustive nature of operative service in dentistry and the limitation of physical ability quickly determines the number of hours each one should sustain continuous effort. To perform good service the condition of the operator must be so maintained that each day, and every hour of each day, he should be in fit condition for efficient service. This cannot be the case if the strain is carried beyond reasonable limits, and he who makes daily demands upon reserved force by extreme effort is in danger of impaired vigor, lessened usefulness, and a shortened life. I have found, notwithstanding the possession of a considerable degree of physical resilience, that seven hours daily effort is the limit of my capacity. The period, however, of satisfactory labor must vary with individuals, depending upon the constitutional force and the intensity of the nervous energy peculiar to each one.

To have the attention confined to the cases in hand, to avoid the waste of time by callers for consultation, and to prevent the nervous friction inevitable to the interruption of important and delicate work, the second proposition was formed. The benefits of this arrangement are beyond calculation, and it works all through the conduct of practice with satisfactory results. The justness of the principle that the engaged patient is entitled to all of the time and the undisturbed attention of the operator is a question of the simplest justice. No other consideration should overweigh this.

The attempt was followed at first by resistance and criticism on the part of some; but the manifest right of the method so appealed to common sense, that as soon as knowledge of the intention was diffused acquiescence became general. The announcement of this arrangement was printed on the appointment cards thus, "that unavoidable calls are received at" a certain hour. The most convenient time for this was found to be after the consultation hour. A practice systematically conducted, however, will not have many unavoidable calls.

Another feature somewhat connected with this subject, and which was adopted before any means were attempted to reduce my practice to system, was the note printed on the engagement cards, reading, "that application for appointments should be made by note." This rule is continued in force. It is intended thus to restrict useless calls. The day is divided into three parts,—the morning, from 9 to 12; the noon, from 12 to 1.30, for examinations, consultations, and rest; the afternoon, until 4.30. This gives six hours for uninterrupted effort, and I venture that if any one will, in a similar manner, resolutely apportion the time, the effect will give the fullest satisfaction. These six hours proceed with complete quietness and without exterior causes of friction. It is also an important consideration that this repose and privacy is especially grateful to the persons under treatment.

The even distribution of appointments was a more difficult problem. The tendency of patients, if left to their own inclinations, when not influenced by dental troubles, is towards having regular attention given to the teeth in the spring and autumn. These periods, therefore, are disposed to be overwhelmed with applications for appointments, with consequent rushing to meet the exigency of the situation. The remainder of the year may not be sufficiently occupied to be stimulating to either body or mind.

Recognizing that the need of dental service by all classes of the community is a certainty, and that the best interest of the patient and the greatest satisfaction of the practitioner is to be secured by regular service rendered to each one, I have for over twenty years maintained a regulated system of notification for renewal of appointments for the care of the teeth. To this the thoughtful and those appreciative of the value of the teeth gave ready acquiescence. Within a few years it became so general that in case any one was overlooked it was usually considered a ground of complaint. In making such renewals I have, however, been careful to give the reasons for this course, and have been thoughtful not to trench upon the liberty of the person, since it is a natural feeling to resent being controlled by others. On the completion of each series of operations, each one, or their representative in the case of children, is informed that a re-examination should be made in from three to six months, as the apparent conditions would seem to require, and generally have accompanied this statement with the request, "Shall this, then, be done?" In such manner this system became thoroughly established. The advantages are so marked in the case of children that only by a similar method can the assurance of the preservation of the teeth from dangerous conditions be secured, and the same principle is applicable in a less degree only to adults.

The details of the method by which these appointments are distributed throughout the year are sufficiently simple. A book is kept, called the monthly index, in which months are printed on the margin instead of the alphabet. When the arrangement for the renewal is effected, the secretary enters the name of the patient at the proper month, with such symbols as may indicate the kind of appointments to be made at the new period.

The average amount of service required by each person when this system is faithfully and continuously carried out is nearly two hours, twice per year; so many requiring less than this as to give opportunity for those needing more to have the required allotment of time. With this apportionment three examination appointments made upon each day, with two subsequent appointments of one hour each, completely fills the available hours; stated in other words, seventy-eight patients per month, on the average, have their wants attended to, and are sufficient to fill all the time.

To meet the requirements of a full practice and to enable this system of management to be conducted, the employment of a secretary as an aid is an absolute necessity. The secretary should be a woman of good education, of refined manners, and of excellent tact. She should have some knowledge of simple book-keeping, be a good penman, capable of writing a courteous note in good language, and should, above all, be thoroughly systematic.

With such an aid dental practice is relieved of many of its burdens.

In addition to carrying out the system indicated, the secretary cares for the orderly arrangement of the instruments, prepares the filling-materials, and in various ways may be instructed to give assistance. All this is not an onerous labor to an orderly woman, and such service offers a function to women who shrink from duties of a more public nature, and who from choice or need of the income desire to be of use. I cannot too strongly commend the use of women secretaries to dental practitioners. Their services tend to the maintenance and development of practice, and are far more satisfactory than aid given by male students, whose intrusions may be a serious hinderance to the growth of practice, since women and children are sensitive to the presence of male assistants.

It will be seen by those of you who will carefully consider this system of governing practice, that while the practitioner appears to be a fixed factor, he is more satisfactorily provided for than if left to the control of circumstances. It is true he is in a great measure bound by what appear to be a series of established obligations to his clientele, but he has greater freedom within those limits than would be the case without similar order. The only hour which is an absolutely binding one is that for consultations. In reference to the other hours, whatever arrangements may be needed for the treatment of individual cases may be provided for, and in case the opening of an hour or the shortening of the day is needed to meet the requirements of health, there need be no reflection that one is neglecting his affairs.

This plan also allows those who are reaching the afternoon of life to lessen their labor and be free to enjoy whatever leisure they may require, and not lose any of the opportunities for practice, or appear to neglect the calls of their patients.

Heretofore I have considered only those features of practice management which concern the efficiency of the practitioner and the best interests of the patient. I have omitted any mention of the finances of the situation. As the nature of dental service is very exhaustive of nervous force and requires much skill to efficiently perform it, and the value and importance of our services to the people is great, we are entitled to high remuneration. But what shall be the initial rate? and how shall this be regulated? are serious and important questions.

The value of all material things is determined by the demand coupled with the ability of the people to pay for them. If the supply too much increases, or the ability of the people becomes impaired, the price diminishes. The same principle is applicable to a less degree only of professional services of every kind. It is the demand which at length determines the question of the income and, to a much less degree than in the case of goods, the ability of the people,—professional services not suffering in this respect in hard times nearly so much detriment as the affairs of the manufacturer or the merchant.

The same opportunity, however, does not exist for the abilities of the professional man becoming known as is the case in general business, and he has to await the occurrence of the fortuitous circumstances combined with the impressions of character which shall make capacity apparent, for the world shrinks from any one who, occupying a professional position, does by any direct effort make pretension of ability, and resists with jealousy the attempt to make capital out of events, unmasking with dreadful cruelty the pretender.

In our specialty, notwithstanding the great demand for our services, it is peculiarly difficult to secure the attention of the public, for the reason that our ministrations are private and confidential, and do not attract general attention like the pleading and counsel of the lawyer or the cures of the general physician.

The thorough performance of duty, however, and the filling of all available time for whomsoever can be found to accept service, even if it has to be done gratuitously, will, if the person possesses the essential qualities, lead him into the upward path. But this consideration does not directly meet the question of income. Shall the beginner who has marked ability charge high or moderate fees? Had he not better accept the latter than restrict the opportunity for full employment by insisting on the former? For in full employment is the road to high income, because this cultivates ability, develops character, and gives the opportunity to bring one's services within the law governing the relations of supply and demand. Many of you must have in your minds men who, because of the knowledge of their own capacity, have restricted their commencement in life by high fees, and others whose high fees being out of relation to the demands for their services, have been badly stranded. The public is intelligent, and associates facts with considerable certainty; it may be slowly, it is true, but nevertheless certainly, and its voice is potent. While the public is jealous, it is also generous, and there is scarcely any limit in reason to the reward which will be given to those deserving its confidence.

The condition of one in full practice may become critical in consequence of the overpressure for service. When all the time is regularly taken and the demand continues, further demand tends to either one or both of two things,—the deterioration of the service or diminishing income. This latter apparently paradoxical condition requires relief. In my experience I have found only one remedy, which is to raise the fees to the new patient. This should be done by notice, and be uniform to this class. The action is essentially the same as that which is daily pursued in the business world; more is asked, and if the public will bid higher a new price is fixed. In the cases before us the result is not immediately reached. Should those subjected to the increased price generally return for treatment, the indication is plain that the service is enhanced in value, whereupon the increased rate can be made general throughout the whole practice. In this manner the fees for dental services can be elevated from time to time without danger and in strict agreement with the laws which govern values, and no one is safe who goes much counter to these regulations. To be guided in the arrangement of charges for dental service by the arbitrary estimate of the value they may have in our mind, or by the assumed ability of the patient to pay, appear to be questionable principles of action. In the one case we are liable to be influenced by personal conceit, in the other we have yielded to the basest of motives, —the desire to have of the wealth of one person more than we cheerfully accept as a just rate from another person.

This brings us to the question, What shall govern the initial fee? What shall be the unit of charges? Shall it be specific for each kind of thing done? Should it be for the time spent? Or should it be an approximate standard of an hour's service?

I have held to the latter, but keeping time as the chief determining element. Given a patient of good quality,—that is, intelligent, healthy, teeth of good structure and not very sensitive,—a standard of an hour's service is easily made up.

Thus to a systematic person, whose mind goes before each action, and who, therefore, wastes no movements, an hour's service becomes a nearly fixed amount of dental labor performed upon the teeth.

The varying conditions and characteristics of patients modifies the amount of service which can be rendered in any given time, and when the physical and moral deficiencies of the patient limit our performance, he becomes responsible for the time expended without relation to the amount of service. Therefore, in these instances, the value of time becomes the cost of the service. In some cases it is impossible to render the half of a standard hour's service in one hour; in such instances the patient is justly responsible for a whole hour.

What, then, becomes of the standard denominated an hour's service? It is only of use to determine the relative condition of the operator. If he fail from want of tone to render an adequate amount of service in any given time, the charge should not be for the time expended, but should be that proportion which in his conscience has an approximate relation to his habitual standard of effort. This may appear complicated in its presentation, but should come within the easy comprehension of all engaged in full practice who are of methodical bent of mind.

There is, however, no rule so efficacious towards controlling the conduct of patients in the fulfilment of appointments, in their endurance of services, and other accommodating considerations, as the knowledge that they are responsible for the time which is being consumed.

It will be observed that this method of being remunerated for service is connected with and is a part of the system of the management of practice which I have presented to you.

It is easily perceived that the application of these principles of management are more applicable to operative dentistry than to a mixed practice. However, in my own, which is almost exclusively operative, what little of it is devoted to dentures is compensated for in the same manner. The different office services connected therewith are charged for at the same rate per hour as for other service, and whatever laboratory expenses are incurred are entered up separately with adequate compensative profit in addition. For operations strictly surgical in their nature, or in orthodontia, the fees should be determined principally by the character and importance of the cases.

These principles regulating compensation for service it must be conceded raises the question from that of "get what one can" to something like a degree of uniformity and more nearly into agreement with right and regard for the interests of others. It also brings the question of fees within the comprehension of the people; and if the above method or a similar one were in general use, I am inclined to believe it would not only remove the reproach which has been cast upon us by departures from what is right and just in these respects, and would, I sincerely think, raise the general compensation for dental service. It is also my impression that such a method would induce the diffusion of patronage among a larger number of those engaged in practice.

These methods are plainly better adapted to the conditions which exist in the larger cities. How far they can be brought into use outside the centres of population is a question which can only be determined by the sentiment which is capable of being developed in each neighborhood.

There may be details of the mode of carrying out this method of management, which will be elucidated during the discussion following the reading, but which could not with propriety be introduced in this paper.

